# Supplementary material for: Validation of a Low-protein Semi-Quantitative Food Frequency Questionnaire
Source: Nutrients. 2022 Apr 12;14(8):1595. doi: 10.3390/nu14081595 (PMC9031454; doi:10.3390/nu14081595)
Supplement: Supplementary file 1 [file nutrients-14-01595-s001.zip › nutrients-1649461-supplementary.pdf]

Table S1: Gender, age and mean z-scores for BMI, weight and height for PKU and control children

	PKU Group	Control Group	P value*
Gender	24M, 26F	24M, 26F	0.99
Mean age years (range)	9.3 (4.2-16.4)	9.3 (4.2-16.6)	0.99
Mean weight z-score (range)	0.76 (−2.0–2.4)	0.66 (−2.3–2.6)	0.54
Mean height z-score (range)	0.26 (−2.4–2.4)	0.60 (−2.1–2.8)	0.14
Mean BMI z-score (range)	0.87 (−1.0–2.9)	0.47 (−3.4–2.7)	0.17

NS=not significant; *Mann Whitney

Table S2: FFQ food items mean g/week FFQ1 vs FFQ2 (PKU and Control)

	No. children consuming FFQ1/ FFQ2 n=50	PKU FFQ1	PKU FFQ 2	Wilcoxon signed rank	Spearman rank correlation	FFQ1/ FFQ2 n=50	Control FFQ 1	Control FFQ 2	Wilcoxon signed rank	Spearman rank correlation
	n	Mean g/week	Mean g/week	p	r	n	Mean g/week	Mean g/week	p	r
Milk, cows	2/3	12.6	15	>0.99	0.82	48/46	1882	1782	0.41	0.71
plant milk	4/7	49	67.2	0.06	0.77	1/1	56	56	0.99	1.0
LP milk	42/40	1421	1509	0.54	0.92					
milk flavouring	6/8	3.3	6.3	0.07	0.52	18/16	14.1	14.3	0.51	0.68
cheese	3/3	3.7	1.8	0.50	0.99	47/45	88.2	87.4	0.75	0.60
soft cheese	5/4	6.6	5.9	0.62	0.89	9/15	6.8	12.8	**0.04**	0.67
LP cheese	31/34	66.4	80.4	0.10	0.66					
soya cheese	1/0	1.2	0	>0.99	-					
dairy desserts	18/17	70	51.6	0.22	0.78	44/41	254	182	**0.0002**	0.72
LP desserts	6/10	21.6	23.4	0.82	0.46					
icecream	12/15	22.8	20.4	0.48	0.60	31/27	46.8	49.2	0.37	0.61
LP ice cream	12/10	34.5	12.6	**0.03**	0.42					
milk based ice lolly	6/7	11.7	9	0.68	0.43	4/6	3.6	6.3	0.38	0.54
fruit based ice lolly	21/20	62.1	58.5	0.51	0.56	14/15	44.1	40.5	0.60	0.45
cream	2/2	1.2	1.8	>0.99	0.47	6/11	6.6	11.4	0.09	0.57
bread	2/3	14.4	19.8	0.50	0.82	49/44	281	242	0.12	0.61
rolls etc	3/1	3	2	>0.99	0.04	30/35	136	142	0.97	0.79
LP bread	45/45	441	421	0.80	0.78					
LP rolls etc	17/24	58	61	0.08	0.39					
GF bread	10/7	19.8	7.8	**0.04**	0.52					
GF rolls etc	4/7	9	9.5	0.98	0.50					
Breakfast cereal - wheat	14/20	23.1	22.4	0.60	0.76	34/30	97.2	73.2	**0.02**	0.73
Breakfast cereal - corn/rice/ oat	29/24	49.5	33.2	**0.04**	0.72	31/35	54.6	49.8	0.43	0.79
Oats/porridge						13/11	90.8	93.8	0.93	0.58
LP breakfast cereal	9/7	9.6	12.9	0.50	0.71					
sweet spreads	26/28	40.2	32.7	0.27	0.83	27/25	17.2	12.5	0.26	0.64
chocolate/nut spread	13/12	24.2	10.8	0.67	0.49	19/19	17.4	15.8	0.74	0.85
LP chocolate spread	9/8	6.2	6.4	>0.99	0.48					
sandwich spread	2/1	2	0.8	0.50	0.71	15/13	15.8	11.4	0.34	0.71
LP savoury spread	3/6	2.4	8.4	0.06	0.45					

butter/ margarine/ oil	47/44	86.9	84.9	0.92	0.64	50/47	50.1	43.6	0.42	0.31
Low fat spread	0/3	0	3.4	0.25	-	6/4	3.3	2.6	0.93	0.32
Pasta/ rice/ couscous	15/14	33.3	34.2	0.80	0.75	49/49	331	334	0.86	0.51
LP pasta/ rice/ couscous	43/43	369	376	0.77	0.81					
tinned pasta	17/9	56	26	**0.003**	0.50	5/8	17.6	27.2	0.86	0.43
LP ready meal / Ready meal	4/4	16	24	0.77	0.20	9/5	24	16	0.52	0.55
potato - boil/ mash/ jacket	42/36	201	189	0.69	0.57	41/44	234	222	0.55	0.50
potato - roast/ chips/ fried	44/46	207	186	0.26	0.51	47/44	176	151	0.34	0.39
potato - processed	28/27	60.3	54.6	0.63	0.67	19/18	25.8	27	0.83	0.94
table sauce	38/37	92.4	87.4	0.38	0.79	44/44	64.4	70.4	0.34	0.55
gravy	27/26	60	52	0.80	0.70	32/31	54.5	42	0.06	0.72
mayonnaise/ dressing	26/26	60.4	42.4	0.25	0.92	13/18	12.4	18.8	**0.007**	0.62
milk sauces	6/4	8.5	9	0.65	0.12	17/16	25	23	0.73	0.50
LP sauce	3/2	8	3	>0.99	0.38					
pasta sauce	32/34	187	152	0.58	0.38	35/39	123	132	0.32	0.51
soup	27/25	238	266	0.77	0.69	14/16	94	80	0.78	0.49
vegetables >100mg Phe/100g	30/34	59.4	73.5	**0.03**	0.68	49/48	263	275	0.48	0.68
vegetables 75-100mg Phe/100g	36/35	113	118	0.73	0.68	39/41	184	202	0.90	0.80
vegetables <75mg Phe/100g	50/49	625	805	**0.01**	0.76	50/50	554	563	0.70	0.59
legumes	15/16	31.8	24.8	0.77	0.85	37/37	154	140	0.80	0.82
fruit	45/44	915	941	0.79	0.74	48/50	939	954	0.62	0.65
dried fruit	17/16	35.4	33.9	0.78	0.35	18/21	31.8	33.9	0.78	0.52
meat						48/48	303	278	0.36	0.57
fish						35/33	78	72.6	0.43	0.81
processed meat/fish						50/49	198	182	0.31	0.49
ham/sandwich slices						33/35	37.7	38.7	0.83	0.76
LP burger/ sausage	23/26	135	168	0.95	0.82					
veg burger/sausage/tofu/quorn	13/15	52.5	36	0.90	0.66	6/8	6	9	0.53	0.50
eggs	0/0	0	0	-	-	29/27	74.5	65	0.49	0.84
Pizza	3/3	8.4	11.2	>0.99	0.65	41/36	107	77.2	**0.02**	0.52
LP pizza	30/31	108	103	0.75	0.69					
meat pie/ pastie	1/0	0.5	0	>0.99	-	26/25	29	30	0.95	0.33
vegetable pie/ pastie	7/6	13	10	0.68	0.25	1/2	1	2	>0.99	0.03
meat curry/ chinese/ lasagne	0/0	0	0	-	-	27/25	126	104	0.44	0.31
vegetable curry/chinese/lasagne	16/15	160	64	0.12	0.47	3/3	12	10	>0.99	0.30
water	47/45	4633	5096	0.73	0.48	43/46	3766	3700	0.62	0.69
fruit juice	28/26	648	520	0.75	0.57	27/22	568	572	0.27	0.75
smoothie	10/11	76	118	0.17	0.58	12/11	112	100	0.54	0.74
sweet drinks	33/32	1176	1686	**0.01**	0.64	17/15	372	305	0.35	0.76
sugar-free drinks	11/9	580	420	0.72	0.43	33/33	2252	2394	0.33	0.75
tea/ coffee	17/19	316	416	0.15	0.79	11/11	184	184	0.78	0.61
hot chocolate powder	3/1	0.9	0.3	0.50	0.57	17/13	9.3	16.2	0.16	0.66
non-dairy creamer	0/2	0	0.9	0.50	-	0/0	0	0	-	-
biscuits	15/15	13.5	22.5	0.21	0.38	43/43	87.2	77.4	0.26	0.66
cake	9/8	20.5	15	0.44	0.54	40/38	123	116	0.81	0.59
cereal bar	20/17	19.6	19.6	0.82	0.77	23/24	31.2	27.2	0.51	0.50
LP biscuit	30/27	58.8	50.1	0.52	0.59					
LP cake	29/30	139	118	0.23	0.66					
LP cereal bar	13/18	38.4	33.2	0.87	0.56					

puddings	1/3	1.6	14.4	0.38	0.04	22/27	81.6	76.8	0.82	0.52
LP puddings	10/9	40	32	0.91	0.24					
jelly	1/1	2	4	>0.99	0.02	10/10	36	38	0.94	0.75
LP jelly	17/23	118	108	0.89	0.71					
chocolate	9/6	12	6.3	0.12	0.65	44/45	76.2	76.2	0.88	0.52
LP chocolate	25/24	34.8	39	0.59	0.58					
gummy sweets	1/3	4.2	7.8	0.50	0.59	26/28	34.8	39.6	0.25	0.73
vegetarian gummy sweets	28/29	53.1	75.9	0.86	0.09					
other sweets	11/15	30.6	29.9	0.99	0.11	10/7	9.6	14.4	0.56	0.69
sugar	19/17	19.0	15.9	0.95	0.61	10/6	5.6	4.35	0.25	0.66
crisps	45/45	72.3	67.2	0.23	0.81	43/42	50.1	48.3	0.17	0.85
nuts/ seeds	0/0	0	0	-	-	9/9	3.8	2	0.18	0.44
LP crisps	4/2	7	2.5	0.63	0.66					
crackers	8/6	5.4	6.2	0.95	0.23	27/26	14.5	11	**0.02**	0.80
LP crackers	16/13	9.2	8.1	0.49	0.74					

n = number respondents consuming that food item; bule: PKU foods only; purple: Control foods only;
-: no; LP=low protein; GF=gluten free; Phe=phenylalanine;

Table S3: FFQ items mean g/week FFQ vs Dietary recalls (PKU and Control)

	No. children consuming food FFQ/ Recall	PKU FFQ average	PKU dietary recalls	Wilcoxon signed rank (p)	Spearman rank correlation (r)	No. children consuming food FFQ/ Recall	Control FFQ average	Control dietary recalls	Wilcoxon signed rank (p)	Spearman rank correlation (r)	Mann Whitney (p) FFQ average PKU vs Control
		Mean g/week	Mean g/week				Mean g/week	Mean g/week			
Milk, cows	3/4	13.8	18.5	0.37	0.87	48/45	1832	1639	0.06	0.79	**<0.0001**
plant milk	7/7	58.0	34.4	0.23	0.54	1/2	56	40	>0.99	0.69	0.06
LP milk	42/38	1465	1105	<0.0001	0.93						
milk flavouring	10/4	4.8	3.8	0.22	0.26	22/17	14.2	12.3	0.52	0.50	0.13
cheese	3/3	2.8	1.9	0.75	>0.99	48/44	87.8	90	0.95	0.39	**<0.0001**
soft cheese	5/4	6.2	12.0	0.19	0.90	16/12	9.8	12.1	0.84	0.29	**0.02**
LP cheese	35/31	73.4	102	0.18	0.77						
soya cheese	1/0	0.6	0	>0.99	-						
dairy desserts	21/21	60.8	86.1	0.16	0.71	46/42	218	238	0.59	0.61	**<0.0001**
LP desserts	12/6	22.5	24.9	0.95	0.01						
icecream	18/7	21.6	15.8	0.24	0.37	35/19	48.0	57	0.91	0.20	0.07
LP ice cream	16/7	23.6	18.2	0.26	0.47						
milk based ice lolly	10/4	10.4	10.2	0.72	0.05	7/15	5.0	40.9	**<0.0001**	0.52	0.36
fruit based ice lolly	27/10	60.3	26.3	**0.01**	0.40	21/7	42.3	26.8	0.13	0.42	**0.03**
cream	3/1	1.5	4.2	0.88	0.03	12/10	9.0	9.1	0.70	0.46	**0.01**
bread	3/9	17.1	28.0	**0.008**	0.65	50/48	261	309	0.06	0.48	**<0.0001**
rolls etc	4/0	2.5	0	0.13	-	35/40	139	204	**0.04**	0.49	**<0.0001**
LP bread	45/45	431	474	0.54	0.64						
LP rolls etc	28/15	59.5	47.7	0.66	0.46						
GF bread	12/7	13.8	20.4	0.67	0.56						
GF rolls etc	8/5	9.3	12.3	0.44	0.79						
Breakfast cereal - wheat	21/16	22.8	27.9	0.20	0.71	37/39	85.2	135	**0.05**	0.58	**0.002**
Breakfast cereal-corn/rice/oat	31/27	41.3	44.4	0.36	0.75	35/24	52.2	40	**0.02**	0.63	0.12
Oats/porridge						16/7	92.3	103	0.62	0.71	
LP breakfast cereal	10/7	11.3	14.7	0.77	0.38						
sweet spreads	30/24	36.5	31.0	0.19	0.78	31/31	14.8	18.5	0.46	0.45	**0.05**
chocolate/nut spread	18/6	17.5	10.1	0.27	0.26	21/16	16.6	16.3	0.69	0.61	0.63
LP chocolate spread	12/3	6.3	5.0	0.06	0.52						

sandwich spread	2/1	1.4	2.8	>0.99	0.71	17/6	13.6	7.4	**0.03**	0.41	**0.002**
LP savoury spread	7/0	5.4	0	**0.02**	-						
butter/ margarine/ oil	48/44	85.9	58.4	<0.0001	0.63	50/46	46.8	46.1	0.74	0.28	**<0.0001**
Low fat spread	3/0	1.7	0	0.25	-	8/1	3.0	0.5	**0.008**	0.38	0.61
Pasta/ rice/ couscous	18/11	33.8	46.7	0.85	0.68	49/44	332	344	0.70	0.31	**<0.0001**
LP pasta/ rice/ couscous	46/34	372	434	0.24	0.74						
tinned pasta	19/4	41	23.4	**0.009**	0.52	10/4	22.4	19.5	0.65	0.43	**0.04**
LP ready meal / Ready meal	7/1	20	5.3	0.14	0.06	10/2	20	5.6	**0.04**	0.41	0.66
potato - boil/ mash/ jacket	43/24	195	145	**0.005**	0.74	47/32	228	154	**0.004**	0.52	0.47
potato - roast/ chips/ fried	47/40	196	231	0.21	0.37	48/38	164	168	0.79	0.32	0.12
potato - processed	32/20	57.5	81.7	0.11	0.73	19/10	26.4	29.5	0.86	0.46	**0.02**
table sauce	39/33	89.9	56.6	**0.0003**	0.68	44/31	67.4	37.3	**0.0008**	0.27	0.55
gravy	30/14	56	44.1	0.13	0.41	34/26	48.3	44.9	0.49	0.47	0.64
mayonnaise/ dressing	28/25	51.4	30.9	**0.04**	0.67	21/16	15.6	13.5	0.39	0.48	**0.02**
milk sauces	9/4	8.8	10.4	0.96	0.25	23/5	24	13.2	**0.02**	0.42	**0.05**
LP sauce	4/3	5.5	2.1	0.44	0.53						
pasta sauce	40/33	170	173	0.84	0.43	45/27	128	97	0.10	0.01	0.75
soup	30/21	252	262	0.21	0.54	20/6	87	40.6	**0.01**	0.52	**0.001**
vegetables >100mg Phe/100g	36/19	66.5	29.2	**<0.0001**	0.61	49/42	269	163	**0.0001**	0.48	**<0.0001**
vegetables 75-100mg Phe/100g	37/21	116	86.5	**0.05**	0.60	44/25	193	71.7	**<0.0001**	0.55	**0.03**
vegetables <75mg Phe/100g	50/48	715	794	0.37	0.42	50/47	558	432	**0.002**	0.62	0.22
legumes	17/13	28.3	26.8	0.47	0.70	38/28	147	158	0.72	0.52	**<0.0001**
fruit	46/43	928	958	0.89	0.75	50/47	946	842	0.07	0.68	0.68
dried fruit	24/17	34.7	30.3	0.17	0.53	26/17	32.9	20	**0.01**	0.60	0.79
meat						48/48	290	314	0.22	0.50	
fish						38/15	75.3	54.0	**0.007**	0.52	
processed meat/fish						50/46	190	256	0.21	0.19	
ham/sandwich slices						37/30	38.2	45.9	0.61	0.56	
LP burger/ sausage	27/22	152	114	0.16	0.66						
veg burger/sausage/tofu/quorn	18/12	44.3	47.4	0.86	0.35	10/3	7.5	13.9	0.80	0.29	**0.03**
eggs	0/1	0	0.4	>0.99	-	31/22	69.8	85.6	0.92	0.48	**<0.0001**
Pizza	4/2	9.8	6.1	0.81	0.31	42/23	92.2	116	0.90	0.09	**<0.0001**
LP pizza	34/12	106	111	**0.04**	0.50						
meat pie/ pastie	1/0	0.3	0	>0.99	-	34/15	29.5	53.4	0.52	0.28	**<0.0001**
vegetable pie/ pastie	11/5	11.5	10.6	0.51	0.31	3/3	1.5	3.4	0.31	0.29	**0.01**

meat curry/ chinese/ lasagne	0/0	0	0	-	-	34/16	115	114	0.54	0.23	**<0.0001**
vegetable curry/chinese/lasagne	21/19	112	159	0.08	0.49	5/3	11	12.6	0.78	0.51	**<0.0001**
water	47/49	4865	3636	0.0005	0.60	47/44	3733	2560	**<0.0001**	0.60	**0.01**
fruit juice	32/25	584	433	0.29	0.52	28/29	570	460	0.31	0.78	0.23
smoothie	14/6	97	72	0.06	0.68	14/6	106	54.0	**0.05**	0.34	0.57
sweet drinks	39/43	1431	1701	0.08	0.65	18/23	339	495	**0.02**	0.64	**<0.0001**
sugar-free drinks	15/7	500	274	**0.005**	0.37	34/33	2323	1781	**0.01**	0.88	**<0.0001**
tea/ coffee	21/15	366	274	0.11	0.77	14/10	184	234	0.34	0.62	**0.02**
hot chocolate powder	3/0	0.6	0	0.25	-	20/9	12.8	8.8	0.08	0.48	**0.002**
non-dairy creamer	2/0	0.5	0	0.50	-	0/0	0	0	-	-	0.49
biscuits	22/16	18	19.8	0.95	0.54	46/44	82.3	115	**0.01**	0.50	**<0.0001**
cake	12/18	17.8	25.1	0.41	0.54	44/44	120	132	0.81	0.17	**<0.0001**
cereal bar	21/9	19.6	10.6	**0.04**	0.60	29/17	29.2	44.1	0.39	0.45	0.34
LP biscuit	34/22	54.5	38.6	0.29	0.33						
LP cake	35/27	129	118	0.40	0.59						
LP cereal bar	21/14	35.8	62.6	0.49	0.45						
puddings	4/1	8.0	1.4	0.25	0.46	32/11	79.2	34.7	**0.0004**	0.43	**<0.0001**
LP puddings	15/8	36	13.8	**0.02**	0.41						
jelly	2/0	3	0	0.50	-	12/10	37	45.5	0.62	0.69	**0.006**
LP jelly	25/8	113	51.1	**0.003**	0.53						
chocolate	10/8	9.2	13.7	0.50	0.65	47/36	76.2	74.1	0.41	0.22	**<0.0001**
LP chocolate	29/13	36.9	23.7	0.06	0.43						
gummy sweets	3/3	6.0	1.6	0.40	0.06	31/14	37.2	11.0	**0.0002**	0.19	**<0.0001**
vegetarian gummy sweets	38/10	64.5	9.1	**<0.0001**	0.21						
other sweets	22/21	30.2	28.1	0.34	0.64	11/10	12.0	9.8	0.89	0.37	**0.002**
sugar	23/21	17.5	17.3	0.54	0.50	11/13	5.0	5.9	0.30	0.56	**0.0004**
crisps	49/42	69.8	99.6	**0.004**	0.64	43/43	49.2	75.1	**0.0009**	0.66	**0.01**
nuts/ seeds	0/0	0	0	-	-	13/3	2.9	1.7	0.14	0.15	**0.003**
LP crisps	4/2	4.8	3.5	0.87	0.72						
crackers	12/12	5.8	15.5	**0.04**	0.69	29/15	12.8	14.8	0.66	0.29	**0.002**
LP crackers	18/8	8.7	10.3	0.59	0.46						

n = number respondents consuming that food; bule: PKU foods only; purple: Control foods only; -: no; LP=low protein; GF=gluten free; Phe=phenylalanine

Table S4: Kappa coefficient statistics FFQ vs Dietary Recalls

	Kappa Score	P-value	Level of Agreement
Energy kJ	0.35	<0.001	Fair
Carbohydrate	0.51	<0.001	Moderate
Protein	0.49	<0.001	Moderate
Fat	0.21	0.03	Fair
Starch	0.52	<0.001	Moderate
Fibre	0.33	<0.001	Fair
Sugars	0.44	<0.001	Moderate

Supplementary S1: FFQ SCRIPT (PKU)

- This questionnaire asks you questions about what you/your child eats.
- I would like you to tell me about all the foods and drinks you/your child eats regularly.
- The questionnaire lists 69 commonly eaten foods, food groups or drinks.
- For each food/food group there is a portion size suggested e.g. teaspoons, tablespoons, bowl, glass, slice.
- A portion size booklet is also provided. This will help you complete the food diary. *(SHOW THEM THE BOOKLET)*
  *(there is a * next to the portions on the FFQ for which there is a picture(s) - for tablespoons or teaspoons refer to the picture on the front of the portion size booklet, for 1 glass (200ml) refer to the Drinks pictures in the portion size booklet)*

- I will ask you to tell me the number of times a 'food portion' is taken each day.
- If you/your child's eats twice the portion size shown, we will count this as 2 portions or if you eat half the portion size shown we will count it as ½ a portion.
- Then I will ask you the number of days a week this amount is eaten.
- If a food is eaten weekly but not daily, tell me how many portions per week.
- If a food is eaten less than once a week we will record it as '0'.
- For food items made up of different ingredients e.g. jam sandwich, I will need to ask you about what ingredients are used e.g. 2 slices low protein bread, 1 teaspoon margarine, 2 teaspoons jam.
- If there are food or drinks you/your child has regularly that are not listed, we can add these in.

General Question (for all entries):

- This is the *(name of heading in table e.g. MILK)* group
- How much/many *(read out food word-for-word from FFQ)* would you/they have a day in terms of *(read out portion size units from questionnaire: i.e.ml/g/slices/pots/tablespoons/teaspoons/pieces/cups/bowls/glasses/serves/packets/exchanges)* (refer to portion size in booklet were applicable). This is *(amount & type of food in picture in portion size booklet)*, how much would you/your child have a day compared with this? More or less than this?
- Do you/your child have this amount every day?
- If you/your child doesn't have it every day how many times a week would you/your child have this much?

(Only put one number in each box NOT a range – ask them to indicate what happens most often e.g. rather than 3-4 times a week specify only 3 or 4. Portion sizes can be in halves or whole numbers).

- Are there any other foods in that group that you/your child eats?

Example 1:
This is the *MILK* group.
How much Milk – full fat, semi-skimmed, skimmed, condensed, soya (include on cereal, in drinks e.g. tea, in foods e.g. mash potato) would you have a day in terms of ml? *(show them the picture of a glass of milk in the portion size booklet)* This is a 200ml glass of milk, so in total how much would you have a day compared with this? More or less than this? If you don't have it every day how many times a week would you have this much?

Example 2:
This is the *PASTA, RICE AND POTATO* group.
How much low protein pasta, noodles, rice, couscous (cooked) would you have a day in terms of grams? *(show them the picture of the low protein pasta in the portion size booklet).* This is 80g and 160g of low protein pasta, how much would you have a day compared with this? More or less than either of these amounts? If you don't have it every day how many times a week would you have this much?

Example 3:
This is the *SAVOURY SNACKS* group.
How much Potato crisps, corn snacks, rice snacks, popcorn would you have a day in terms of a small packet (15-25g)? Would you have more or less than one packet a day? Or if not every day, how many times a week?

ADDITIONAL QUESTIONS
Read questions out but show them the responses so they can indicate their answers. Indicate that they need to answer based on <u>what usually happens on most days</u>.
E.g.
Q.1. If a certain meal/snack is not eaten every day, but more often than not, then still tick the box. If not, leave it blank.
Q.3. If salt is <u>usually</u> added to food then tick yes.
Q.8/10. If protein exchanges/protein substitute are not always eaten at a particular time of day, only tick the box if it is more often than not.

Supplementary S2: FFQ SCRIPT (CONTROL)

- This questionnaire asks you questions about what you/your child eats.
- I would like you to tell me about all the foods and drinks you/your child eats regularly.
- The questionnaire lists 69 commonly eaten foods, food groups or drinks.
- For each food/food group there is a portion size suggested e.g. teaspoons, tablespoons, bowl, glass, slice.
- A portion size booklet is also provided. This will help you complete the food diary. *(SHOW THEM THE BOOKLET)*
  *(there is a * next to the portions on the FFQ for which there is a picture(s) - for tablespoons or teaspoons refer to the picture on the front of the portion size booklet, for 1 glass (200ml) refer to the Drinks pictures in the portion size booklet))*

- I will ask you to tell me the number of times a 'food portion' is taken each day.
- If you/your child's eats twice the portion size shown, we will count this as 2 portions or if you eat half the portion size shown we will count it as ½ a portion.
- Then I will ask you the number of days a week this amount is eaten.
- If a food is eaten weekly but not daily, tell me how many portions per week.
- If a food is eaten less than once a week we will record it as '0'.
- For food items made up of different ingredients e.g. jam sandwich, I will need to ask you about what ingredients are used e.g. 2 slices white bread, 1 teaspoon margarine, 2 teaspoons jam.
- If there are food or drinks you/your child has regularly that are not listed, we can add these in.

General Question (for all entries):

- This is the *(name of heading in table e.g. MILK)* group
- How much/many *(read out food word-for-word from FFQ)* would you/they have a day in terms of *(read out portion size units from questionnaire: i.e.ml/g/slices/pots/tablespoons/teaspoons/pieces/cups/bowls/glasses/serves/packets)* (refer to portion size in booklet were applicable). This is *(amount & type of food in picture in portion size booklet)*, how much would you/your child have a day compared with this? More or less than this?
- Do you/your child have this amount every day?
- If you/your child doesn't have it every day how many times a week would you/your child have this much?

(Only put one number in each box NOT a range – ask them to indicate what happens most often e.g. rather than 3-4 times a week specify only 3 or 4. Portion sizes can be in halves or whole numbers).

- Are there any other foods in that group that you/your child eats?

Example 1:
This is the *MILK* group.
How much Milk – full fat, semi-skimmed, skimmed, condensed, soya (include on cereal, in drinks e.g. tea, in foods e.g. mash potato) would you have a day in terms of ml? *(show them the picture of a glass of milk in the portion size booklet)* This is a 200ml glass of milk, so in total how much would you have a day compared with this? More or less than this? If you don't have it every day how many times a week would you have this much?

Example 2:
This is the *PASTA, RICE AND POTATO* group.
How much pasta, noodles, rice, couscous (cooked) would you have a day in terms of grams? *(show them the picture of the pasta in the portion size booklet).* This is 80g and 160g of pasta, how much would you have a day compared with this? More or less than either of these amounts? If you don't have it every day how many times a week would you have this much?

Example 3:
This is the *SAVOURY SNACKS* group.
How much Potato crisps, corn snacks, rice snacks, popcorn would you have a day in terms of a small packet (15-25g)? Would you have more or less than one packet a day? Or if not every day, how many times a week?

ADDITIONAL QUESTIONS
Read questions out but show them the responses so they can indicate their answers. Indicate that they need to answer based on <u>what usually happens on most days</u>.
E.g.
Q.1. If a certain meal/snack is not eaten every day, but more often than not, then still tick the box. If not, leave it blank.
Q.3. If salt is <u>usually</u> added to food then tick yes.
Q.8/10. If protein exchanges/protein substitute are not always eaten at a particular time of day, only tick the box if it is more often than not.

Supplementary S3: SCRIPT FOR 24 DIETARY RECALL (PKU)

TO BE COMPLETED BY THE RESEARCHER BY INTERVIEW (FACE-TO-FACE OR TELEPHONE) WITH SAME PARENT EACH TIME (5-10Y CHILD) OR WITH THE CHILD ONLY (11-16Y)

We want to find out what you/your child eats and drinks on a typical day.

This includes all food, drinks, snacks and supplements eaten at home or outside the home including school (if not known can we ask your child?).

We also need to know how much was eaten – e.g. number of slices, tablespoons, teaspoons, cups, grams, ounces, ml or exchanges.

Only include the amount of food actually eaten, not what was offered.

We also need to know:

- brand names of products if known
- types of food (e.g. low protein, gluten-free or regular; type of milk: full fat/semi-skim/skim; type of spread: low fat/regular; type of bread: wholemeal/ white bread/half 'n half)
- how foods were cooked (e.g. fried, grilled, baked, boiled).
- If you can provide recipes or estimate quantities for home cooked foods.

We want to record everything you/your child had to eat or drink yesterday.

Was yesterday a normal day? i.e. no parties, celebration day, illness.

If yesterday was not normal can you tell me about the day before (or the last 'normal' day you can recall).

What day of the week was this? *(circle the day at the top of the diary page)*.

Starting with the beginning of the day, approximately what time was the first food or drink taken? *(record estimated time e.g. 8am)*.

What food/drink was taken first?

- **Brand**, **type**, **low protein/gluten-free,** **how cooked/prepared**, **amount** eaten, **recipe** if applicable?

What other food/drink was taken at that time?

- **Brand**, **type**, **low protein/gluten-free,** **how cooked/prepared**, **amount** eaten, **recipe** if applicable?

REPEAT ABOVE QUESTIONS UNTIL ALL FOOD/DRINK CONSUMED AT THAT TIME IS LISTED

Check: did you/they have any:

- cereal/toast/low protein milk (**Brand**, **type**, **low protein/gluten-free**, how **cooked/prepared**).
- spreads on bread/toast? (**Brand**, **type**, **amount)**.
- drinks e.g. drink/juice/water/milk/low protein milk? What type of cup, glass, mug or carton?
- anything added to food e.g. salt, seasoning, butter
- protein substitute (**Brand**, **amount** taken).

Any other comments about that meal? *(e.g. how they felt, eaten later/earlier than usual).*

REPEAT THE FOLLOWING SCRIPT FOR EACH SEPARATE MEAL/SNACK CONSUMED

When is the next time you/they have something to eat/drink? (record estimated time).

What food/drink was taken?

- **Brand**, **type**, **low protein/gluten-free**, **how cooked/prepared**, **amount** eaten, **recipe** if applicable?

What other food/drink was taken at that time?

- **Brand**, **type**, **low protein/gluten-free**, **how cooked/prepared**, **amount** eaten, **recipe** if applicable?

Did you/they have any:

- bread/vegetables/fruit/yoghurt/crisps/biscuits/pudding/other snacks/low protein milk (**Brand**, **type**, **low protein/gluten-free**, how **cooked/prepared**).
- spreads on bread/toast? (**Brand**, **type**, **amount)**.
- drinks e.g. juice/water/milk/low protein milk? What type of cup, glass, mug or carton?
- anything added to food e.g. salt, seasoning, butter
- protein substitute (**Brand**, **amount** taken).

Do you/they have any bedtime drinks or any drinks overnight?

Any other comments about that meal? e.g. how they felt, eaten later/earlier than usual.

Now I am going to read back what you have told me for each meal and snack, and if there is anything missing or if anything is incorrect please let me know.

READ THROUGH EACH MEAL/SNACK CHECKING FOOD/DRINK, QUANTITY, BRAND, TYPE (LOW PROTEIN/GLUTEN-FREE/REGULAR), COOKING METHOD. CHECK THAT ALL PROTEIN SUBSTITUTE AND LOW PROTEIN MILK IS RECORDED.

---

24 HOUR DIETARY RECALL

SUBJECT NO: _______

INITIALS: _______

DATE: _______

Please circle the correct day: **Mon/ Tue / Wed / Thu / Fri / Sat / Sun**

**Time of day**	**Amount eaten**<br>g / oz / ml / slices / tbsp / tsp	**Type of food / drink / protein substitute**<br>Include brand names, method of cooking, recipes, type (e.g. low protein, skim, low fat)	**Comments**

EXAMPLE:

Please circle the correct day: **Mon / Tue / Wed / Thu / Fri / Sat / Sun**

**Time of day**	**Amount eaten** g / oz / ml / slices / tbsp / tsp	**Type of food / drink / protein substitute** Include brand names, method of cooking, recipes, type (e.g. low protein, skim, low fat)	**Comments**
e.g.<br>8am	174ml<br>Small bowl<br>100ml	PKU Cooler 20<br>Low protein cereal (Loprofin strawberry flakes)<br>Prozero	Breakfast later than usual
11am	1<br>47g<br>1 glass	Apple<br>Yoghurt (Petit Filous)<br>Sugar free squash	
1.00-1.30pm	2 slices<br>2 tsp<br>2 tsp<br>16g packet<br>174ml	Low protein bread (Mevalia)<br>Butter<br>jam<br>Quavers<br>PKU Cooler 20	Only ate half
3.30pm	2<br>200ml	Low protein biscuits (wafers)<br>Sugar free squash	
5.30-6.00pm	1<br>1 ½ exchanges<br>60g<br>2 tbsp<br>174ml<br>1 glass	Low protein sausage<br>Potato chips (oven baked)<br>Broccoli<br>Carrots<br>PKU Cooler 20<br>water	
7pm	1	Mevalia fruit bar	

Supplementary S4: SCRIPT FOR 24 DIETARY RECALL (CONTROL)

TO BE COMPLETED BY THE RESEARCHER BY INTERVIEW (FACE-TO-FACE OR TELEPHONE) WITH SAME PARENT EACH TIME (5-10Y CHILD) OR WITH THE CHILD ONLY (11-16Y)

We want to find out what you/your child eats and drinks on a typical day.

This includes all food, drinks, snacks and supplements eaten at home or outside the home including school (if not known can we ask your child?).

We also need to know how much was eaten – e.g. number of slices, tablespoons, teaspoons, cups, grams, ounces or ml.

Only include the amount of food actually eaten, not what was offered.

We also need to know:

- brand names of products if known
- types of food (e.g. type of milk: full fat/semi-skim/skim; type of spread: low fat/regular; type of bread: wholemeal/ white bread/half 'n half)
- how foods were cooked (e.g. fried, grilled, baked, boiled).
- If you can provide recipes or estimate quantities for home cooked foods.

We want to record everything you/your child had to eat or drink yesterday.

Was yesterday a normal day? i.e. no parties, celebration day, illness.

If yesterday was not normal can you tell me about the day before (or the last 'normal' day you can recall).

What day of the week was this? *(circle the day at the top of the diary page).*

Starting with the beginning of the day, approximately what time was the first food or drink taken? *(record estimated time e.g. 8am).*

What food/drink was taken first?

- **Brand**, **type**, **how cooked/prepared**, **amount** eaten, **recipe** if applicable?

What other food/drink was taken at that time?

- **Brand**, **type**, **how cooked/prepared**, **amount** eaten, **recipe** if applicable?

REPEAT ABOVE QUESTIONS UNTIL ALL FOOD/DRINK CONSUMED AT THAT TIME IS LISTED

Check: did you/they have any:

- cereal/toast (**Brand**, **type**, **how cooked/prepared**).
- spreads on bread/toast? (**Brand**, **type**, **amount**).
- drinks e.g. juice/water/milk? What type of cup, glass, mug or carton?

- anything added to food e.g. salt, seasoning, butter

Any other comments about that meal? *(e.g. how they felt, eaten later/earlier than usual).*

REPEAT THE FOLLOWING SCRIPT FOR EACH SEPARATE MEAL/SNACK CONSUMED

When is the next time you/they have something to eat/drink? (record estimated time).

What food/drink was taken?

- **Brand**, **type**, **how cooked/prepared**, **amount** eaten, **recipe** if applicable?

What other food/drink was taken at that time?

- **Brand**, **type**, **how cooked/prepared**, **amount** eaten, **recipe** if applicable?

Did you/they have any:

- bread/vegetables/fruit/yoghurt/crisps/biscuits/puddings/other snacks (**Brand**, **type**, **how cooked/prepared**).
- spreads on bread/toast? (**Brand**, **type**, **amount**).
- drinks e.g. juice/water/milk/low protein milk? What type of cup, glass, mug or carton?
- anything added to food e.g. salt, seasoning, butter

Do you/they have any bedtime drinks or any drinks overnight?

Any other comments about that meal? e.g. how they felt, eaten later/earlier than usual.

Now I am going to read back what you have told me for each meal and snack, and if there is anything missing or if anything is incorrect please let me know.

READ THROUGH EACH MEAL/SNACK CHECKING FOOD/DRINK, <u>QUANTITY</u>, <u>BRAND</u>, <u>TYPE</u>, <u>COOKING METHOD</u>.

---

24 HOUR DIETARY RECALL

SUBJECT NO: _______

INITIALS: _______

DATE: _______

Please circle the correct day: **Mon/ Tue / Wed / Thu / Fri / Sat / Sun**

**Time of day**	**Amount eaten**<br>g / oz / ml / slices / tbsp / tsp	**Type of food / drink / protein substitute**<br>Include brand names, method of cooking, recipes, type (e.g. skim, low fat)	**Comments**

EXAMPLE:

Please circle the correct day: **Mon / Tue / Wed / Thu / Fri / Sat / Sun**

**Time of day**	**Amount eaten** g / oz / ml / slices / tbsp / tsp	**Type of food / drink / protein substitute** Include brand names, method of cooking, recipes, type (e.g. low protein, skim, low fat)	**Comments**
e.g.<br>8am	200ml<br>Small bowl<br>100ml	Orange juice<br>cereal (Special K)<br>Milk (Skim)	Breakfast later than usual
11am	1<br>47g<br>1 glass	Apple<br>Yoghurt (Petit Filous)<br>Sugar free squash	
1.00-1.30pm	2 slices<br>2 tsp<br>2 tsp<br>16g packet<br>200ml	bread (white)<br>Butter<br>jam<br>Quavers<br>water	Only ate half
3.30pm	2<br>200ml	biscuits (chocolate digestive)<br>Sugar free squash	
5.30-6.00pm	2<br>15<br>60g<br>2 tbsp<br>200ml<br>1 glass	sausages<br>Potato chips (oven baked)<br>Broccoli<br>Carrots<br>Sugar free squash<br>water	
7pm	1 square	Flap jack	
